# Infraorbital Foramen and Pterygopalatine Fossa Location in Dry Skulls: Anatomical Guidelines for Local Anesthesia

**DOI:** 10.1155/2017/1403120

**Published:** 2017-12-19

**Authors:** Omar Masabni, Maha Ahmad

**Affiliations:** ^1^Department of Periodontology and Dental Hygiene, University of Detroit Mercy School of Dentistry, Detroit, MI, USA; ^2^Department of Biomedical and Diagnostic Sciences, University of Detroit Mercy School of Dentistry, Detroit, MI, USA

## Abstract

**Purpose:**

The aim of the study was to locate the infraorbital foramen (IOF) in relation to the infraorbital margin (IOM) for proper injections of local anesthetics in skull specimens. Another aim was to determine the depth of needle penetration into pterygopalatine fossa through the greater palatine canal (GPC).

**Materials and Methods:**

102 skull halves were used to measure the distances between (1) IOF and IOM and (2) IOF and alveolar ridge of maxilla at second premolar. Needles were inserted and bent at a 45° angle, passing through the GPC at the level of hard palate. The depth of the tip of needle emerging out of GPC into pterygopalatine fossa was measured.

**Results:**

The mean distance between IOF and IOM was 6.46 ± 1.57 mm on the right side and 6.74 ± 1.72 mm on the left. The mean distance between IOF and alveolar bone process of the maxilla at second premolar was 29.07 ± 3.58 mm on the right side and 29.39 ± 3.78 mm on the left. The mean depth of penetration of the needle into the pterygopalatine fossa was similar on both sides.

**Conclusions:**

Proper identification of IOF and pterygopalatine fossa is of great significance during local anesthesia injections, due to their close proximity to vital anatomic structures.

## 1. Introduction

Nerve blocks, used to anesthetize the maxillary incisors, canine, and premolars, target the infraorbital nerve (ION) [1], a branch of the maxillary division (V2) of the trigeminal nerve. The ION enters the orbit through the inferior orbital fissure and then transverses the infraorbital canal located under the floor of the orbit [2]. While the ION is in the canal, it provides sensory innervations to the maxillary anterior teeth (anterior superior alveolar nerve), premolars (middle superior alveolar nerve), and associated gingiva. The ION exits the orbit through the infraorbital foramen (IOF) where it becomes a terminal branch, dividing into three branches that provide sensory innervations to the upper lip (superior labial nerve), lateral side of the nose (lateral nasal nerve), and lower eyelid (inferior palpebral nerve).

The greater palatine nerve (GPN) is a branch of the pterygopalatine ganglion that descends downward into greater palatine canal (GPC) and emerges into the oral cavity at the level hard palate through the greater palatine foramen.

The IOF is located in close proximity to vital anatomical structures; therefore proper identification of its location during regional block anesthesia is highly recommended [3, 4]. However, this can be challenging due to its anatomical variation. Several previous studies attempted to determine the location of the IOF by the use of different reference points during ION block anesthesia. One of the constant reference points used is the maxillary second premolar [2, 5–8], as it lies in the same sagittal plane as the IOF.

A previous research paper used GPC to give a maxillary nerve block in the superior aspect of the pterygopalatine fossa [2], while another research study inserted the needle at a lower level inside pterygopalatine fossa [9].

The aim of this study was to locate the IOF in relation to the infraorbital margin (IOM) and the alveolar bone process of the maxilla at the second premolar for proper injections of local anesthetics, as well as to determine the depth of needle penetration into the pterygopalatine fossa through GPC to avoid serious complications following nerve block anesthesia.

## 2. Materials and Methods

The materials used in this study consisted of 102 halves of 51 dry intact adult skulls that were examined in the Gross Anatomy Laboratory at the University of Detroit Mercy School of Dentistry. The age and gender of the skulls were not available.

### 2.1. Measurement of IOF in relation to IOM and Maxillary Alveolar Ridge at the Second Premolar

**Figure 1:**
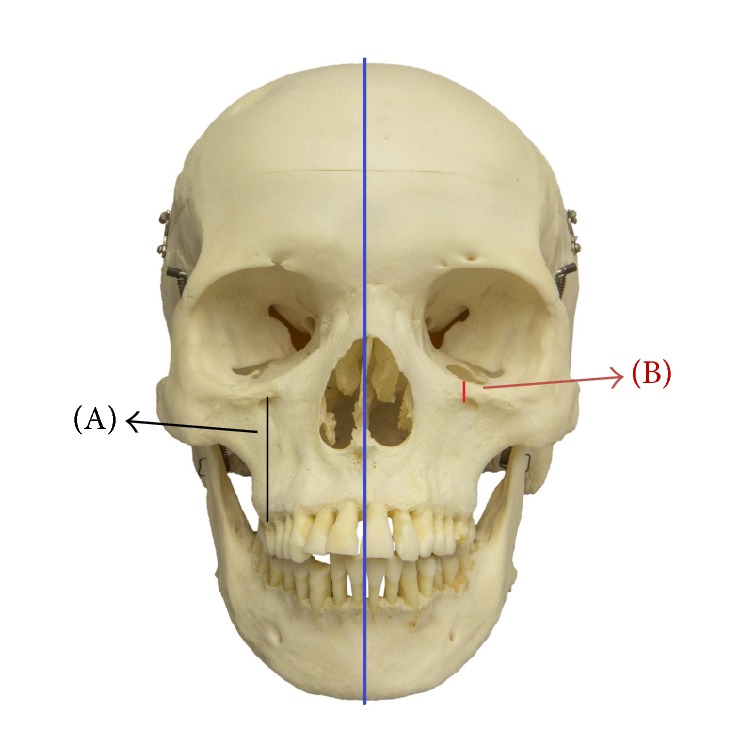
It shows the frontal view of a human skull. Distances were calculated as shown in the figure. A parallel longitudinal line to the midsagittal plane from (A) the superior border of infraorbital foramen (IOF) to the maxillary alveolar ridge and (B) from the superior border of the IOF to the infraorbital margin (IOM).

Superior border of IOF directly overlying the IOF was the main reference point from which vertical measurements to IOM and maxillary alveolar ridge at the second premolar were recorded. Therefore skulls that had fractures in the IOF, IOM, and GPC were excluded. The vertical distance was measured from the highest point of the IOF to the IOM, parallel to sagittal plane and perpendicular to Frankfurt plane. Another vertical distance measured was the distance from the most superior border of IOF to the maxillary alveolar ridge at the second premolar, parallel to sagittal plane and perpendicular to Frankfurt plane (Figure 1). The vertical distances were measured using a digital caliper (Mitutoyo 500-196-30 0-6′′) of 0.01 mm precision.

### 2.2. Measurement of Needle Penetration Depth into the Pterygopalatine Fossa

**Figure 2:**
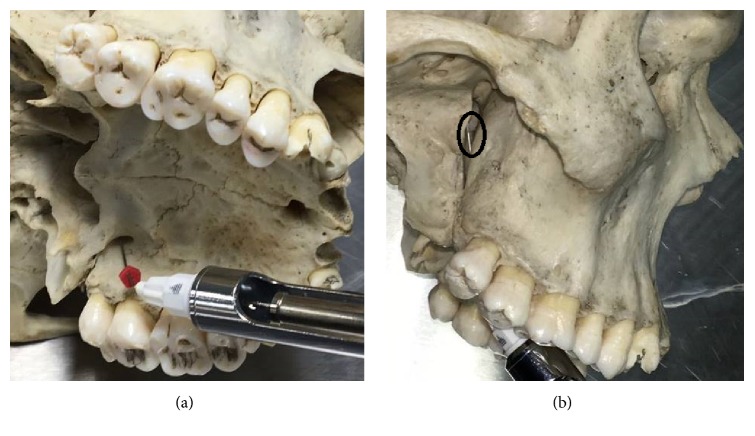
(a) shows insertion of needle bent at a 45° angle through greater palatine foramen into greater palatine canal. (b) shows the needle tip (circle) as it projects out of the greater palatine canal into pterygopalatine fossa.

A 25-gauge, 32 mm needle was bent at 45° angle immediately after the hub, at the beginning of the shaft, and a rubber stopper was introduced into the needle. Then, the needle was inserted through the GPF and once the needle tip approached the pterygopalatine fossa and became visible, the rubber stopper was adjusted at the level of the hard palate and the depth of needle penetration was measured (Figure 2).

## 3. Results

There were no statistically significant differences between right and left sides of the skull for IOF-IOM, IOF-Maxillary alveolar process at the second premolar and GPC measurements (Table 1). The median, mode, and range were comparable for each set of measurements in both halves. The distance between the IOF and maxilla was more than 4 times greater than the distance between the IOF and IOM. The depth of GPC was significantly smaller than the distance between the IOF and the maxilla. Demographic information, such as age and gender, of the skull was not available; thus, comparing our results based on this data was not performed.

## 4. Discussion

In this study, we determined the location of the IOF and its distance from two important anatomical structures, the IOM and the alveolar process of the maxilla in 51 human skulls from a gross anatomy laboratory. Superior border of IOF was our main reference point as it is prominent and locating this anatomic landmark is easily identified on patients by dentists. Therefore, the results in our study are clinically applicable and dentists can use these references as an approximation during nerve block anesthesia.

Previous studies used the piriform aperture as a reference to locate the IOF, which dentists cannot palpate clinically on patients, thus making it an unreliable measure to approximate the IOF location [14, 15]. Therefore, we used the superior border of IOF as our main reference point for identifying the location of the IOF, as this is a prominent feature that can be easily distinguished on patients during dental procedures. We found that the distance from IOM to either structure was not significantly different between the different hemispheres of the skull. Furthermore, there was little variation among the samples tested: 6.6 ± 1.65 mm from the IOF to IOM and 29.23 ± 1.65 mm from IOF to alveolar process of the maxilla at the second premolar.

Previously published literature examining the distance between IOF and IOM showed results comparable to our study (Table 2). However, slight discrepancies in the results of these studies might be attributed to the population studied, type of specimens (dry skulls or images), and the method of measurement used to acquire the data.

Using the IOF as a reference due to its ease of identification by the practitioner, as well the distances measured in this study as a general guide are clinically applicable for use as an approximation for ION block anesthesia.

An additional aim was to determine the depth of needle penetration into pterygopalatine fossa through GPC to help dentists avoid serious complications following regional block anesthesia. The hard palate approximately forms a 60° angle with GPC [16] and the patient is usually seated in a semi-reclined position with the mouth opened and head tilted back. Therefore, to prevent further penetration of the needle deep into the pterygopalatine fossa, we suggested bending the needle at 45° angle. Once the tip of the needle approached the pterygopalatine fossa and became visible, the rubber stopper was adjusted at the level of hard palate and the depth of needle penetration was measured. The clinical significance of this technique is to avoid injury to nearby nerves and blood vessels during injections of local anesthetic solution. In a previous study conducted by Mercuri [2] the author suggested bending the needle at a 30° angle before inserting it into the most superior border of the pterygopalatine fossa. However, with this technique, the tip of the needle was in close proximity to the main trunk of maxillary artery and pterygopalatine ganglion. Branches from the pterygopalatine ganglion supply the nasal cavity and roof of the oral cavity and spread to the ocular regions [1] which might increase the risk of anesthetizing the ocular nerves. The technique followed in this paper is more favorable than Mercuri's [2] technique, as it prevents further progression of the needle beyond the bend which in return will avoid the incidence of injury and simultaneously ensures that profound anesthesia is obtained in that area with least postoperative complications.

In this study, the age of the skulls and their gender were not available; thus the effect of gender on the location of IOF could not be evaluated. Another limitation is that the maxillary alveolar process may resorb as a result of aging, following tooth extraction or due to periodontal disease.

## 5. Conclusion

The average distance between the IOF and IOM in dry intact human skulls was 6.6 ± 1.65 mm and the average distance between IOF and alveolar bone process of the maxilla at second premolar was 29.23 ± 1.65 mm. The average depth of penetration of needle into GPC was 17.41 ± 2.69 mm. We recommend bending the needles at a 45° angle to facilitate entry into GPC. Without this bend, further penetration of the needle might lead to serious complications following local anesthesia injections. Therefore, proper identification of the location of IOF and pterygopalatine fossa is highly recommended during regional block anesthesia and surgical procedures, due to the close proximity to vital anatomic structures.

## Figures and Tables

**Table 1:** Anatomical measurements taken by a digital caliper in mm. ^*∗*^SD: standard deviation.

Measurements	Right				Left				Mean
	Mean ± SD^*∗*^	Median	Mode	Range	Mean ± SD^*∗*^	Median	Mode	Range	Total
IOF-IOM	6.46 ± 1.57	6	6	4–11	6.74 ± 1.72	6	6	3–12	6.6 ± 1.65
IOF-Max	29.07 ± 3.58	28.5	30.5	20.5–36.5	29.39 ± 3.78	29	31	21–36	29.23 ± 1.65
GPC	17.56 ± 2.88	17	18.5	11.5–25.5	17.25 ± 2.51	17	16.5	11.5–23.5	17.41 ± 2.69

**Table 2:** Studies comparing locations of IOF to IOM sorted by date.

Studies	Measurements (mm)	Number of skulls, ethnic background	Measurements done on
1993, Hindy and Abdel-Raouf [10]	6.10	(i) 30 adult skulls	(i) Dry skulls
		(ii) 15 adult human cadavers, Egypt	(ii) Radiographs (panoramic)
1995, Chung et al. [5]	8.60	124 skulls, Korea	Photographs
1998, Silva [11]	6.80	100 human skulls	Skulls
1999, Canan [12]	Females: 8.30	45 cadavers	Skulls
	Males: 10.90		
2000, Aziz et al. [6]	Females: 7.80	47 cadavers	Skulls
	Males: 8.50		
2001, Kazkayasi et al. [8]	7.19	Cadavers	Microscopically
2002, Karakaş et al. [13]	6.70	31 Caucasians	Skulls
2009, Macedo et al. [14]	6.37	295 human skulls	Skulls
2011, Singh [15]	6.16	55 Indian skulls	Skulls
2015, Aggarwal et al. [3]	6.32	67 dry adult skulls	Skulls
2017, this study	6.60	51 human skulls	Skulls
